# HOW CAN NEW TECHNOLOGIES HELP REDUCE ABSENTEEISM IN PEDIATRIC CONSULTATION?

**DOI:** 10.1590/1984-0462/2020/38/2018313

**Published:** 2020-01-13

**Authors:** Neliane da Silva Bueno, Andrea Maciel de Oliveira Rossoni, Elisângela Aparecida da Silva Lizzi, Tony Tanous Tahan, Tatiane Emi Hirose, Herberto José Chong

**Affiliations:** a Universidade Federal do Paraná, Curitiba, PR, Brazil.; b Universidade Tecnológica Federal do Paraná, Cornélio Procópio, PR, Brazil.

**Keywords:** Tuberculosis, Child, Treatment refusal, Absenteeism, Tuberculose, Criança, Recusa do paciente ao tratamento, Absenteísmo

## Abstract

**Objective:**

To identify the most effective form of contact, as a possible intervention to reduce absenteeism in consultations of children with suspected or confirmed pulmonary tuberculosis.

**Methods:**

A randomized clinical trial was conducted with prospective data collection, between March 2017 and February 2018. Patients were randomized into three groups to be reminded about the appointment: telephone contact, SMS or WhatsApp, or no intervention. A convenience sample was obtained, with a significance level of 5%.

**Results:**

78 children were included, with a median age of four years old (zero to 14); 59.0% of them were in treatment for a latent infection and 6.4% had active tuberculosis. Among the 78 children, 74.4% lived in Curitiba (Sourhern Brazil); 62.8% lived with both parents; 38.5% of the parents had formal employment and 47.4% of the mothers were housewives; 50.8% of the fathers and 55.7% of the mothers had more than nine years of schooling. In 78.2% of the families, per capita income was up to 0.5 minimum wages; 27.3% were enrolled in social programs; 28.2% lived in homes provided by the government. There was a total of 238 interventions made: 85 (35.7%) by telephone contact, 78 (32.8%) by text message (WhatsApp was 97.2% of these) and 75 (31.5%) had no further contact. There was no statistical difference among the sociodemographic and cultural characteristics studied. The absenteeism rate was 24.0% and the abandonment rate was 16.7%. Giving a reminder to the patient’s guardian prior to the consultation, regardless of the intervention (p=0.021) and specifically by WhatsApp message (p=0.032) was associated with no absenteeism, though it was not associated with abandonment of the treatment.

**Conclusions:**

Using new tools, such as WhatsApp, to remind guardians of appointments reduces absenteeism. Consequently, it may lead to a reduction in abandoning treatment and it may improvetreatment outcome of children with a tuberculosis infection or disease.

## RESUMO

**Objetivo:**

Identificar a forma mais efetiva de contato, como possibilidade de intervenção, para diminuir o absenteísmo em consultas de crianças com suspeita ou com tuberculose pulmonar.

**Métodos:**

Ensaio clínico randomizado com coleta de dados prospectiva, entre março de 2017 e fevereiro de 2018. Os pacientes foram aleatorizados em três grupos para relembrar a consulta: contato telefônico; mensagens curtas (SMS) ou *WhatsApp*; e nenhuma intervenção. Amostra de conveniência, com nível de significância de 5%.

**Resultados:**

Incluídas 78 crianças, mediana de idade quatro anos (zero a 14); 59,0% em tratamento para infecção latente e 6,4% com tuberculose ativa; 74,4% moravam em Curitiba, Paraná; 62,8% residiam com ambos os pais; 38,5% dos pais possuíam emprego formal e 47,4% das mães eram do lar; 50,8% dos pais e 55,7% das mães possuíam mais de nove anos de estudo; em 78,2% das famílias a renda *per capita* foi de até 0,5 salário mínimo; 27,3% estavam inscritas em programas sociais; e 28,2% residiam em casa cedida. Foram 238 intervenções: 85 (35,7%) por contato telefônico, 78 (32,8%) por mensagem de texto (*WhatsApp* 97,2%) e 75 (31,5%) sem nenhum contato adicional. Nas características sociodemográficas e culturais estudadas não houve diferença estatística. O absenteísmo foi de 24,0% e o abandono, de 16,7%. Lembrar o responsável previamente à consulta, independente da intervenção (p=0,021) e especificamente por mensagem por *WhatsApp* (p=0,032) foi associado ao não absenteísmo, porém não associado ao abandono.

**Conclusões:**

O uso de novas ferramentas, como o aplicativo *WhatsApp,* pode reduzir o absenteísmo, diminuir a possibilidade de abandono no seguimento e melhorar o desfecho do tratamento de crianças com tuberculose, seja a infecção ou a doença.

## INTRODUCTION

Tuberculosis is an infectious disease that is considered to be one of the leading causes of death in underdeveloped and developing countries, which makes it a public health problem. According to the World Health Organization (WHO), in 2016, tuberculosis was one of the top ten causes of death from infectious disease worldwide, higher than the human immunodeficiency virus (HIV/AIDS). In 2016, there were 10.4 million new cases of tuberculosis, and children under 15 years old represented 6.9% of this total.^1^

In 2014, during the World Health Assembly, a new global strategy for tackling tuberculosis as a public health problem was approved. The goal of abolishing the disease by the year 2035 is in place. It is represented by an incidence coefficient of less than 10/100 thousand inhabitants, through the strategy End TB, which aims at “a world free of tuberculosis”. As such, the Brazilian Ministry of Health began to construct the National Plan to End Tuberculosis, which outlines mechanisms to achieve this goal, as well as defines indicators for monitoring the progress of the actions employed. The national plan is structured on three pillars, which are focused on patient-centered prevention and integrated care, bold policies and support systems, and intensified research and innovation.^2^ In this way, children should be prioritized through health policies aimed at them, especially concerning the services involved in preventing children exposed to tuberculosis from abandoning treatment.

The aim of this study was to identify the most effective form of contact, as a possibility of intervention, in order to reduce absenteeism in consultations with children who have pulmonary tuberculosis or suspected pulmonary tuberculosis.

## METHOD

The study included a randomized clinical trial with prospective data collection of children in outpatient tuberculosis follow-up treatment at a tertiary center in Curitiba, Paraná, in the south of Brazil. Inclusion criteria were: aged between zero and 14 years old; consulted between March 2017 and February 2018; in treatment for active or latent tuberculosis or possible tuberculosis; parents or guardians have a cell phone or they gave the contact information of a person close to them; signed free and informed consent form and the consent form for children over 12 years old. There were no exclusion criteria.

A convenience sample was performed. To determine the minimum sample size, the calculation was performed with a confidence interval of 95% (95% CI) and a sampling error of 5%, so that a necessary sample of 131 interventions was considered.^3^ During the outpatient consultations, patients and their guardians were invited to participate in the study. During inclusion in the research, a questionnaire was completed with the clinical, social, economic and cultural characteristics of the child and his or her family. It was established with the guardian which telephone numbers were best in order to get in contact, and if they preferred to receive short text messages (SMS) or WhatsApp messages (voice or text message).

At subsequent return visits within 24 to 48 hours prior to the appointment, subjects were randomly assigned to one of the intervention groups, which corresponded to how the parents or guardians would be reminded of the scheduled appointment, or not. The information sent to the designated contact was made in a standardized manner, reinforcing the date, time and place of the appointment. When more than one individual was in the same household and scheduled for an appointment, they were placed in the same group. The interventions were:

- **Message group:** SMS or WhatsApp (audio or text message), as chosen in the interview, sent to the parents, guardians or designated person’s mobile phone.
- **Telephone group:** Phone call to parents, guardians or the designated person. Up to three calls were made to the reported numbers.
- **Group without an additional intervention:** No prior contact was made with the parents or guardians.

For all individuals, the date, time and place of the appointment were recorded on a hospital card, according to the pre-established routine of the institution. Children were randomly and independently allocated to any of the intervention groups at each outpatient appointment, and the parents or guardians did not previously know how they would be contacted. Children who returned to their appointments more than once during the research period were randomly allocated again and considered to be a new intervention. Thus, the number of interventions was greater than the number of patients.

For children who did not show up to consultations, a telephone call was made to identify the reasons that caused them to miss, and to reschedule the consultation. Children who had two consecutive missed appointments were considered to have quit treatment and were no longer included in new interventions, but continued routine outpatient follow-up.

The data obtained by the researcher through the data collection instrument were entered into a spreadsheet (Microsoft Excel^®^), checked, validated and exported for statistical analysis. Quantitative variables were described in terms of mean and standard deviation, while qualitative variables were described by frequency and percentage. The data were submitted to Pearson’s chi-square test to verify the association between the variables. In these analyzes, a significance level of 5% was considered and adjustments were obtained using the STATA^®^ (version 12.0, StataCorp LP, Texas, United States) software.

The study was approved by the Human Research Ethics Committee of the *Complexo Hospital* of the *Universidade Federal do Paraná* (CAAE: 61854216.5.0000.0096; Report No. 1,948,281).

## RESULTS

During the study period, between March 2017 and February 2018, 78 children were treated at the outpatient clinic and included in the study. Of these, 61 (78.2%) were new patients and 17 (21.8%) were already in outpatient follow-up; five (31.2%) had already missed previous consultations, and had an average of 1.4 missed appointments (±0.55). None of the families refused to participate in the study.

In this case series, 58 patients (74.4%) lived in Curitiba, 41 (52.3%) were male, with a median age of four years old (and ranging from 0 to 14 years old). Forty-nine patients (62.8%) lived with both parents, 30 (38.5%) fathers had formal employment and 37 mothers (47.4%) were housewives. Regarding education, 30 fathers (50.8%) and 39 mothers (55.7%) had more than nine years of schooling. Two fathers and one mother did not have any education. In 54 (69.2%) households, the family income was up to two minimum wages and in 61 (78.2%) households, the per capita income was up to 0.5 minimum wages; 43 (55.1%) families were not enrolled in federal government social programs and 22 (28.2%) lived in a provided house.

The history of contact with tuberculosis was identified in 74 (94.9%) individuals, and in 39 (50.0%), the source was the parents or stepfather. Among people who had drug addicts in the home, 13 (16.7%) respondents reported having family members who were using some kind of licit or illicit drug. Eight (10.3%) were alcoholics, and of these, five (6.4%) were the father. In 11 cases (14.1%), the person with a chemical dependency was the source.

Mothers brought their children when they were included in the survey, and answered the study questionnaire in 56 (71.8%) cases. Regarding how the mothers would like to be reminded of the consultation, 31 (39.7%) respondents preferred a text message (SMS or WhatsApp), 25 (32.1%) preferred the hospital card and 22 (28.2%) preferred being contacted by telephone. In the survey, when the participants selected the option to receive a message, 65 (83.3%) opted for a *WhatsApp* text, 0 for an audio message and 13 (16.7%) for an SMS text message. With regard to the contact telephone number, in 64 (82.0%) cases, it was a parent’s number.

Regarding knowledge about tuberculosis, 55 (70.5%) guardians reported understanding the disease, 62 (79.5%) believed that the child was not ill and 40 (51.3%) were not afraid of the disease.

Throughout the research, the 78 patients underwent 238 interventions on how to reinforce the scheduling of appointments (by telephone, text message or just the reminder on the hospital card). Regarding the interventions performed, 85 (35.7%) were contacted by telephone, 78 (32.8%) received a text message, 68 (28.6%) through WhatsApp text. Seventy-five (31.5%) did not receive any additional contact. The amount and type of intervention varied for each patient, as described in Table 1.

**Table 1:** Quantity and type of interventions performed on patients seen at a tertiary outpatient clinic in southern Brazil.

Type of intervention	Number of interventions							
	1 n (%)	2 n (%)	3 n (%)	4 n (%)	5 n (%)	6 n (%)	7 n (%)	Total n (%)
Telephone	34 (14.3)	15 (6.3)	16 (6.7)	8 (3.4)	2 (0.8)	2 (0.8)	2 (0.8)	85 (35.7)
Message	25 (10.5)	25 (10.5)	15 (6.3)	8 (3.4)	3 (1.3)	1 (0.4)	1 (0.4)	78 (32.8)
None^a^	19 (8)	25 (10.5)	14 (5.9)	11 (4.6)	2 (0.8)	4 (1.7)	0 (0)	75 (31.5)
Total	78 (32.8)	65 (27.3)	45 (18.9)	27 (11.4)	13 (5.5)	7 (2.9)	3 (1.2)	238 (100)

^a^No additional interventions performed.

In the telephone contact intervention, an average of 1.6 calls were made, and in 17 (20.0%) of those, the guardians did not answer the three attempts. Of the calls answered, 48 (70.6%) were the guardian who answered, and 38 (55.9%) were the mother. There was no association between those who answered the phone and those who had come to the consultation. Furthermore, if we consider telephone contact to be only those who answered the call as an intervention, there was no reduction in absenteeism (p = 0.087).

During this period, in 181 (76.1%) interventions, the children attended the scheduled appointments, and in 57 interventions (24.0%), they missed them. The reasons given by the child’s guardians regarding the absences are described in Table 2, and the frequency of the consultations is described in Table 3. Thirteen (16.7%) children were considered to have missed the appointment, 43 (55.1%) were discharged from the hospital, and the others continued in follow-up treatment. Upon assessing the type of intervention and the frequency of consultations, the following were found: when the intervention was a text message, there was a reduction in absenteeism, with a relative risk (RR) of 1.22 and a 95%CI of 1.01–1.47, unlike when a phone call was made (RR = 1.16; 95% CI 0.96–1.41).

**Table 2:** Causes of absenteeism in patients seen at a tertiary outpatient clinic in southern Brazil.

Cause	n (%)
Forgetting about the appointment or confusion around the scheduled date	18 (31.6)
Lack of money to pay for transportation	12 (21)
Missing the bus	6 (10.5)
Sick child	4 (7)
Guardian had difficulty missing work	4 (7)
Guardian was sick	3 (5.3)
Guardian thought the child was not sick	3 (5.3)
Guardian went out of town	2 (3.5)
Child custody changed	2 (3.5)
Lack of parental commitment	2 (3.5)
Child was being treated in another service area	1 (1.8)

**Table 3:** Frequency of consultations in patients seen at a tertiary outpatient clinic in southern Brazil.

Frequency of consultations	n (%)
Came to all of the scheduled appointments	40 (51.3)
Missed one appointment	21 (27.0)
Missed two consecutive appointments	8 (10.3)
Missed one appointment and the guardian asked to be transferred to a different service area	3 (3.8)
Missed two intermittent appointments	3 (3.8)
Missed three appointments, two of which were consecutive	3 (3.8)

New patients in the outpatient clinic missed appointments in 52 (30.2%) consultations, while established patients missed five (7.6%), regardless of whether they received an intervention or not (p <0.001). And when absenteeism was evaluated in patients who received any type of intervention, four (7.3%) established patients missed, while 28 (25.9%) new patients missed appointments (p = 0.006).

In order to assess possible biases of influence from previous interventions on the same patient, an assessment was performed separating patients from whether or not they had received any intervention, which type of intervention they had received, and the frequency with which they showed up to consultations. (Table 4 -- Comparative data for all types of intervention, and interventions that occured during the different times the patient was in the outpatient clinic are not described in the table because they are not statistically significant). The protective intervention for absenteeism was to send a message in the first intervention (RR = 1.45; 95%CI 1.05–2.02), regardless of whether the patient was new (p=0.008) or had already received followed up treatment at the outpatient clinic (p=0.006).

**Table 4:** Comparison between the interventions performed to give reminders about the consultations for patients treated at a tertiary outpatient clinic in southern Brazil.

Intervention	Came (%)	Did not come (%)	RR (95%CI)	p-value
Any intervention (n = 238)				
Yes	131 (55.0)	32 (13.5)	1.21 (1.01–1.44)	0.021^a^
No	50 (21.0)	25 (10.5)		
Telephone contact regardless of if they answered the call (n = 160)				
Yes	66 (41.3)	19 (11.9)	1.16 (0.96–1.41)	0.121^a^
No	50 (31.2)	25 (15.6)		
Telephone contact only if they answered the call (n = 160)				
Yes	54 (33.7)	14 (8.7)	1.18 (0.98–1.42)	0.092^a^
No	62 (38.8)	30 (18.8)		
Message (n=170)				
Yes	77 (45.3)	18 (10.6)	1.22 (1.01–1.47)	0.032^a^
No	50 (29.4)	25 (14.7)

RR: relative risk; 95% CI: 95% confidence interval; ^a^chi-square test.

We compared the type of intervention performed with the various characteristics researched: sex, education level of the parents or guardians, who brought the child to the medical appointment, who was the patient’s guardian, whether or not it was a new patient, having already missed an appointment before joining the research, if the guardian understood the diagnosis or was afraid of the disease, if he or she thought the child was sick or not, if he or she had a family member that had already been treated for tuberculosis, if he or she had a drug addiction, if they received a government subsidy, family income, whether parents or guardians were employed, and whether they were from Curitiba. In all of these conditions, the intervention groups were homogeneous and these characteristics did not interfere in whether or not they missed the consultations.

## DISCUSSION

The present study indicates that the most effective intervention to reduce absenteeism in appointments scheduled at a specialized outpatient clinic for childhood tuberculosis is to send a reminder about the consultation through the WhatsApp application. A reduction of this absenteeism may contribute to an improvement in the treatment of tuberculosis, especially considering that the age group analyzed is the group with the highest risk for the illness.

In this study, most parents had a mobile phone or a smartphone, showing that, even among low-income families, they are widely used. Among those who did not have a smartphone, it did not prevent them from participating in the research, as they indicated the number of another family member. When the number is up to date, Whatsapp can be a tool to more easily locate patients. However, it is important for this number to be updated frequently.

When comparing the interventions performed in the general group between contact through the telephone and through text message, the second method was more effective, with an absenteeism rate of 8.5%. For the telephone contact, it was 11.9%. No studies have been found in the literature on the use of WhatsApp messages to reduce absenteeism in consultations, only on the use of SMS text messages. According to a 2011 study in Saudi Arabia, message reminders were effective in reducing absenteeism rates in outpatient appointments, although they varied by specialty. Additionally, there was great patient satisfaction with the messaging service, indicating that the messages could be used to increase interaction with patients.^4^ In this study, the initial messages were used only to give reminders about the date, time, and place of the appointment. However, the authors suggest that this type of technology should also be used to inform patients about the importance of returning to treatment, therapy, and other general information about the disease and treatment, allowing for the possibility to instruct the patient about his or her disease, as well as about follow-up. This type of situation often occurred in this study with subsequent message exchanges with family members. However, it is important to reinforce the need to take care with regard to ethical-legal issues. Message exchanges should not replace face-to-face consultations or guide diagnoses or therapeutic approaches.

Sending reminders to patients via telephone or SMS is considered to be a form of telemedicine, as it involves distance and is a type of technology that contributes to the healthcare process. There is evidence that reminders have a positive effect on non-attendance rates. A systematic review showed that an improvement in the baseline absence rate of 39% and 29%, respectively, can be expected when using manual and automated reminders.^5^

In this study, the interaction established between health professionals and guardians was successful. In several situations, parents interacted through messages to clarify questions about exams and medications and to request that the appointment be rescheduled, when for some reason they could not attend on the scheduled date. Therefore, the messages became a link between healthcare professionals and guardians, especially through the WhatsApp application. Short text messages can serve as a simple reminder to take medication, which addresses barriers related to adherence, such as forgetfulness and lack of social support.^6^ Text messaging has the advantage of being efficient and considerably less invasive to people’s daily lives in comparison to phone calls.^7^

It was observed that performing any type intervention was more effective for getting the patient to return than just writing down the appointment on a hospital card. In all of the interventions performed, messages were more effective than phone calls.

The outpatient clinic’s routine before this study was to remind guardians about the previously scheduled appointment through a phone call. In another study, with this same population, it was shown that returning patients believed they knew more about tuberculosis and missed the follow-up less often.>^8^Thus, it is believed that these factors decreased absenteeism in the group of returning patients by comparing them with new patients, who were less attached to the outpatient professionals.

It is worth noting that in several situations, when contacting the guardians of the child, they reported that they had forgotten the previously scheduled date and possibly, if there was no intervention, the child would have missed the consultation. Implementing joint actions and the use of new tools are of fundamental importance to minimize the absenteeism rate and offer a more humanized and personalized service.^9^

When it was impossible to contact the guardians, primary health care units and tutelary councils were set into motion, so that guardians were informed about the rescheduled consultation. As for working with networks, we must understand that it is fundamental in articulating complementary actions, which aim to facilitate users’ access to public health services.^10^

During the study period, the rate of absenteeism at consultations was high; and the main cause was forgetting about the consultation. In Rio de Janeiro, in a study conducted between 2005 and 2009, missing treatment rates of children undergoing treatment for a latent infection were 25.3%.^11^ In this study, it was not possible to relate absenteeism and/or stopping treatment to the use of medication or to the length of the follow-up period. Absenteeism may delay a diagnosis and, consequently, the start of treatment, which may interfere with the child’s health. These absences ultimately increase the length of the follow-up period, and thus reduce the number of vacancies available for other patients awaiting outpatient consultation. In a public health context, the discussion about absenteeism in outpatient consultations should be emphasized, since it directly wastes structural and financial resources available to citizens, in addition to incurring social costs.^12^ Thus, interventions that give reminders about consultations with new tools, such as the WhatsApp application, should be encouraged. In the present day of multitasking, this type of action helps patients, because it creates more of a bond, creates a more welcoming atmosphere, and improves the outcomes of follow-ups.

This study, despite showing a reduction in absenteeism with regards to remembering the consultation, did not establish the same association with quitting treatment, probably due to a limitation in the study design. All patients underwent several different interventions and there was no control group that did not have an intervention. Thus, at some point, everyone received some kind of reminder, which must have caused this lack of association. Other limitations were the convenience sample, which could have led to a selection of patients with a higher or lower risk of absenteeism, but we believe that this did not influence the result, because during the study period all children who sought out outpatient care were included.

With the high absenteeism rate found, effective measures must be taken to reduce these rates. Thus, when dropout rates do not increase, personal and institutional costs of care are reduced and outcomes are improved. It should be emphasized that the main cause of absenteeism was forgetfulness. When asked ahead of time, most guardians preferred to be reminded of the scheduled appointment. In this study, no other factors were associated with absenteeism.

Children have age-specific characteristics that distinguish them from other groups and place them in a position of vulnerability and dependence. In this sense, all favorable actions to reduce absenteeism and contribute to the treatment of diseases are in accordance with the Child and Adolescent Statute, which guarantees the doctrine of comprehensive protection, where children are understood to be full citizens, who should be given priority in protection, as they are people in physical, mental, moral, spiritual and social development.^13^

Finally, this research sought to give visibility to an extremely relevant topic, such as childhood tuberculosis, and the possibilities of using tools that can reduce absenteeism in follow-up consultations. World goals are aimed at eradicating this disease, and further research with new intervention populations should be conducted to confirm these findings.

## References

[1] World Health Organization [homepage on the Internet] (2017). Global tuberculosis Report 2017.

[2] Brazil - Ministério da Saúde (2017). Secretaria de Vigilância em Saúde. Departamento de Vigilância das Doenças Transmissíveis. Brasil livre da tuberculose. Plano nacional pelo fim da tuberculose como problema de saúde pública.

[3] (2018). Prática Clínica [homepage on the Internet].

[4] Youssef A, Alharthi H, Khaldi O Al, Alnaimi F, Alsubaie N, Alfariss N (2014). Effectiveness of text message reminders on nonattendance of outpatient clinic appointments in three different specialties: A randomized controlled trial in a Saudi Hospital. J Taibah Univ Med Sci.

[5] Hasvold PE, Wootton R (2011). Use of telephone and SMS reminders to improve attendance at hospital appointments: a systematic review. J Telemed Telecare.

[6] Oren E, Bell ML, Garcia F, Perez-Velez C, Gerald LB (2017). Promoting adherence to treatment for latent TB infection through mobile phone text messaging: study protocol for a pilot randomized controlled trial. Pilot feasibility study.

[7] Kaplan WA (2006). Can the ubiquitous power of mobile phones be used to improve health outcomes in developing countries?. Global Health.

[8] Bueno NS (2018). Principais fatores e medidas para prevenção do abandono do tratamento de crianças com tuberculose doença ou infecção latente [master’s thesis].

[9] Monken SF, Moreno RC (2015). Use of control alerts as a tool for the loyalty of the customers of pediatrics in a public clinic. RAHIS.

[10] Canelada HF, Levorato CD, Corte RI, Diniz EE (2014). Redução do absenteísmo através da gestão da agenda e do trabalho em rede. Anais do Congresso Internacional de Humanidades & Humanização em Saúde.

[11] Mendonça AM, Kritski AL, Land MG, Sant’Anna CC (2016). Abandonment of treatment for latent tuberculosis infection and socioeconomic factors in children and adolescents. PLoS One.

[12] Bittar OJ, Magalhães A, Martines CM, Felizola NB, Falcão LH (2016). Absenteísmo em atendimento ambulatorial de especialidades no estado de São Paulo. BEPA.

[13] Brazil – Presidência da República [homepage on the Internet] (2018). Lei 8.069 de julho de 1990. Dispõe sobre o Estatuto da Criança e do Adolescente e dá outras providências.

